# The Study of Crystallization Behavior, Microcellular Structure and Thermal Properties of Glass-Fiber/Polycarbonate Composites

**DOI:** 10.3390/polym15061546

**Published:** 2023-03-21

**Authors:** Xinchao Wang, Yapeng Sun, Jiale Hu, Lan Wu, Tie Geng, Yonggang Guo, Chenhao Zhao, Binbin Dong, Chuntai Liu

**Affiliations:** 1 School of Mechanical & Electrical Engineering, Henan Provincial Engineering Research Centre of Automotive Composite Materials, Henan University of Technology, Zhengzhou 450001, China; 2 National Engineering Research Center for Advanced Polymer Processing Technologies, Zhengzhou University, Zhengzhou 450002, China

**Keywords:** supercritical CO_2_ microinjection, polycarbonate composite, crystallization behavior, thermal properties

## Abstract

Polycarbonate (PC) foam is a versatile material with excellent properties, but its low thermal stability limits its application in high-temperature environments. The aim of this study was to improve the thermal stability of PC foam by adding glass fibers (GF) and to investigate the effect of GF on PC crystallization behavior and PC foam cell morphology. This study was motivated by the need to improve the performance of PC foams in various industries, such as construction, automotive, and medical. To achieve this goal, PC/GF composites were prepared by extrusion, and PC/GF composite foams were produced using a batch foaming process with supercritical carbon dioxide (SC-CO_2_) as the blowing agent. The results showed that the addition of GF accelerated the SC-CO_2_-induced crystallization stability of PC and significantly increased the cell density to 4.6 cells/cm^3^. In addition, the thermal stability of PC/GF foam was improved, with a significant increase in the residual carbon rate at 700 °C and a lower weight loss rate than PC matrix. Overall, this study highlights the potential of GF as a PC foam reinforcement and its effect on thermal and structural properties, providing guidance for industrial production and applications.

## 1. Introduction

In recent years, the preparation of microcellular foams and their applications have received increasing attention due to their lightweight nature and excellent toughness of microcellular foam products. Polycarbonate (PC) is a highly versatile material with excellent properties such as transparency, mechanical strength, dimensional stability, heat resistance, and insulation, making it widely used in various industries. However, PC also has some limitations, such as poor fatigue resistance, low notched impact strength, high melt viscosity, and weak resistance to chemical media, which restrict its application potential [1]. To overcome these challenges and enhance the material’s performance, microcellular foaming technology can be utilized during the processing of PC. This technology improves impact toughness and fatigue life, reduces density and thermal conductivity, and expands the range of potential applications for PC [2]. PC foam has been used in a variety of applications, including insulation panels for the construction industry, interior trim for the automotive industry, and prosthetics and orthotics for medical devices, among others [3,4,5,6]. By incorporating microcellular foaming technology, PC’s application potential can be further broadened, providing enhanced durability and toughness, making it suitable for use in more demanding applications across multiple industries. When selecting a blowing agent as the main agent for the preparation of polymeric microporous materials, the chemical blowing agent is left in the final product. The traditional physical blowing agents, such as chlorofluorocarbons (CFCs) and hydrochlorofluorocarbons (HCFCs), could deplete the ozone layer. Hydrofluorocarbons (HFCs) aggravate the greenhouse effect, and hydrocarbons (HCs) are hazardous because of their high flammability [7]. Therefore, the physical blowing agent, Supercritical carbon dioxide (SC-CO_2_), is superior to the chemical and other physical foaming methods because of its operational controllability and environmental friendliness [8]. Polymers commonly used in the market, such as polystyrene (PS) [9,10,11], polymethyl methacrylate (PMMA) [12,13], polylactic acid (PLA) [14,15], polycarbonate (PC) [16,17], polyethylene (PE) [18], polypropylene (PP) [18,19,20], polyamide 6 (PA6) [21], polyvinyl chloride (PVC) [22], and polyurethane (PU) [23], have been reported in detail for the preparation of microcellular foams using SC-CO_2_. In addition, SC-CO_2_ induces the crystallization of polymers, and calculations and analyses related to crystallization have been extensively studied [24,25].

In addition to foaming polymers, nanomaterials or fibers have been added to adjust the microporous foaming behavior and the final bubble structure to determine the relative density and macroscopic properties of the microporous material. Studies on PC composite foaming have focused on multi-walled carbon nanotubes (CNTs) [26,27], nanoclay [28], and graphene nanoplatelets (GnP) [29,30]. Lai et al. [26] reported that CNTs acted as cell nucleation during foaming, which increased the cell density and promoted the generation of PC crystals. Hu et al. [28] noted that the addition of nanoclay could reduce the PC crystallization time and form a large number of crystals in a short period of time. Gedler et al. [30] found that the incorporation of graphene into nanocomposites had a higher CO_2_ loss rate and a higher diffusion coefficient. However, the study on glass fiber (GF) composite PC foaming has been rarely reported. Wang et al. [31] found that the addition of GF decreased the size of the cells and increased the density of the cells using GF and PLA composite foaming.

However, there is no detailed result on the effect of the addition of glass fiber on the blister morphology and crystallization behavior, or on the improvement in the mechanical and thermal properties of PC due to the addition of GF. In this study, the crystallization behavior and foaming behavior of PC and PC/GF composite foam under different conditions have been investigated. The molecular structure of the prepared PC/GF composites was analyzed and compared with that of pure PC. The crystallization properties, crystalline properties, cellular morphology, and thermal stability of PC and composite foams prepared using SC-CO_2_ as a physical blowing agent were also investigated. The effect of GF loading and immersion time in SC-CO_2_ on the complex melting behavior of the prepared foams was emphasized. This study of glass fibers as composites for polycarbonate foams provides insights that may improve the mechanical and thermal properties of the material. This research could contribute to the development of more advanced and efficient polycarbonate foam materials for various industrial applications such as automotive, aerospace, building, and construction.

## 2. Materials and Methods

### 2.1. Materials

Polycarbonate (2805, density 1.2 g/cm^3^, glass transition temperature 149 °C, melting index 17.5 dg/10 min) was supplied from Bayer Corporation, Leverkusen, Germany. Foaming agent carbon dioxide is obtained from Changte Gas Co., Ltd., Zhengzhou, China, with a purity of 99.99%. Glass Fiber (TX-PC-3.0, density 2.7 g/cm^3^, silane coupling agent treatment, monofilament diameter 11 um, length 3.0 mm) was obtained from Taixin Co., Ltd., Guangzhou, China.

### 2.2. Pretreatment

PC and GF were pre-dried in a vacuum oven (ZKXF Shanghai establish instrument Co., Ltd., Shanghai, China) at 110 °C for 8 h. Then a single-screw extruder (SY-6216-A Dongguan Shiyan Precision Instrument Co., Ltd., Dongguan, China) was used for primary mixing and pelletizing, with joining sections of 245 °C, 265 °C, and 280 °C, respectively. Hot pressing of pure PC was performed with the mixture using a plate vulcanizing machine (XLB-D/Q Zhengzhou Xinhe Machine Manufacturing Co., Ltd., Zhengzhou, China), with a hot pressing temperature of 245 °C and a hot pressing time of 20 min. The hot pressing sheet was cut into 20 mm × 20 mm × 2 mm using a table saw (S1-2-3 Henan Fengrui Co., Ltd., Nanyang, China). The foaming experiment was carried out on a square sheet. The loading rate of GF in the PC matrix was 5.10, 15 wt%, and the code of the composite filled with GF was specified as PC/GF5, PC/GF10, and PC/GF15. The reaction kettle was raised to different temperatures (140 °C, 160 °C, 180 °C, 200 °C), carbon dioxide (CO_2_) at different pressures (8 MPa, 9 MPa, 10 MPa), and held for different times (30 min, 60 min, 90 min, 120 min, 180 min, 240 min, 300 min, 480 min). Then the pressure in the reaction kettle was quickly released within 3 s, and the sample was removed and shaped in cold water to complete the foaming.

### 2.3. Characterization and Measurement

Crystallization properties (melting behavior) of foamed samples were evaluated using a differential scanning calorimeter (DSC 8000 PerkinElmer Instruments Co., Ltd., Waltham, MA, USA), with scanning at 10 °C/min from 50 °C to 250 °C. The rheological properties of the samples were evaluated using a flat plate rotational rheometer (HAAKE MARSIII Thermo Fisher Scientific Corporation, Waltham, MA, USA) at a temperature of 240 °C and test conditions in the frequency range 100–0.1 rad/s with a 1% strain scan. The microporous structures of PC and PC/GF foamed samples were observed using a scanning electronic microscope (SEM) (FEI Quanta 450 FEG Thermo Fisher Scientific Corporation, United States). The accelerating voltage used was set at 3 KV. The thermal properties of PC and PC/GF composites were carried out on the STA-8000 Thermo-gravimetric Analysis (TGA) (PerkinElmer Instruments Co., Ltd., USA) heated from 50 to 700 °C with a heating rate of 20 °C/min. The nitrogen flowrate was 20 mL/min. The Fourier transform infrared spectroscopy (FTIR, SP3 PerkinElmer Instruments Co., Ltd., USA) was used to detect the composition of flammable gases coming from TGA testing.

## 3. Results and Discussion

### 3.1. FTIR Analysis of PC and PC/GF

**Figure 1:**
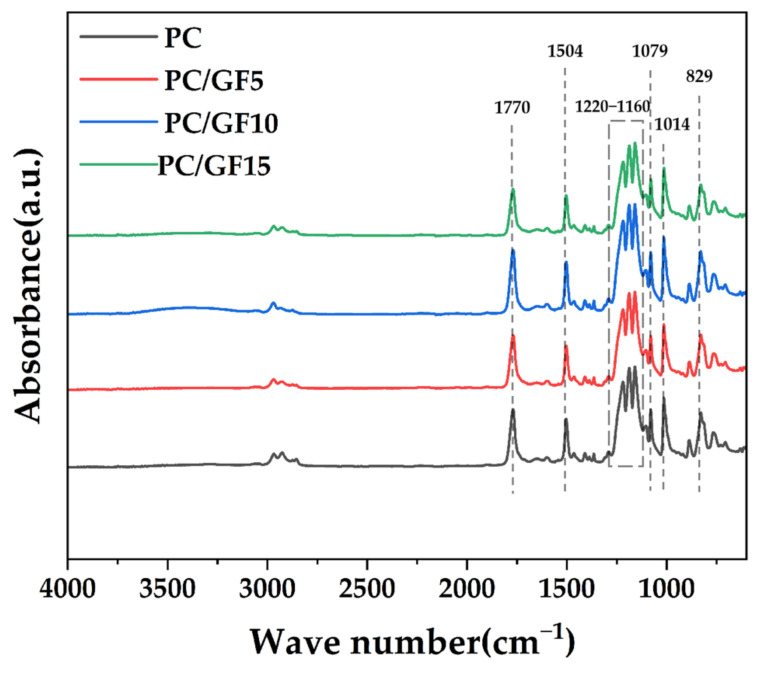
FTIR diagrams of PC/GF composites and PC.

In order to study the effect of glass fiber addition on the composition of PC/GF composites, the infrared spectra of pure PC and PC/GF composites were tested, as shown in Figure 1. 1770 cm^−1^ is the stretching vibration peak of C=O in carbonate, and 1504 cm^−1^ is the stretching vibration peak of C=C double bond in the benzene ring. The C-O-C stretching vibration values are 1219, 1187, and 1159 cm^−1^, and the in-plane bending of benzene ring=CH is 1079 and 1014 cm^−1^, and the out-of-plane bending of the benzene ring=CH is 829 cm^−1^. These are the classical polycarbonate characteristic peaks [32]. Comparing the IR curves of pure PC and PC/GF composites, it was found that the addition of GF did not change the characteristic peaks in the pure PC material. Therefore, the mixing of PC and GF is just a physical mixing and does not change the molecular structure properties of PC. Kuram et al. [33] tested GF-reinforced PC/PBT composites and came to a similar conclusion that the addition of GF did not change the characteristic peaks of the PC/PBT composites. Hacioglu et al. [34] obtained similar IR spectral test results for GF and carbon fibers (GF) on PC.

### 3.2. Rheological Properties of PC and PC/GF

**Figure 2:**
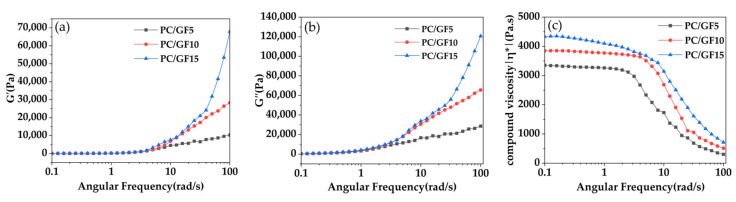
Energy storage modulus G′ (**a**), loss modulus G″ (**b**) and composite viscosity η* (**c**) of PC/GF composites versus frequency.

To investigate the impact of GF content on the rheological properties of PC/GF composites, rotational rheological testing was conducted at a temperature of 240 °C. Figure 2 displays the dependence of the energy storage modulus, loss modulus, and composite viscosity on the angular frequency. The energy storage modulus and loss modulus remained nearly constant at the low frequency range of 0.1–1 rad/s. Subsequently, as the frequency increased to 1–100 rad/s, the energy storage modulus and loss modulus rose with the addition of GF content. This result suggests that the presence of GF restrains the activity of PC molecular chains, leading to a decrease in the material’s flowability.

As depicted in Figure 2c, a typical Newtonian viscosity plateau region was observed in the low frequency range, followed by a shear thinning phenomenon as the frequency increased. Notably, regardless of the frequency, an apparent increase in composite viscosity was observed with the addition of GF content, indicating that the inclusion of GF enhances the melt strength of PC. Similar results were obtained by Knutsson et al. [35] at 290 °C, where the GF-reinforced PC melt exhibited a higher viscosity than the unfilled melt. This study on the rheological properties of PC/GF composites provides valuable insights into the impact of GF on the foaming behavior of PC/GF composites.

### 3.3. Crystallization Behavior of PC and PC/GF Composites

#### 3.3.1. Effect of GF Content on Crystallization Behavior

**Figure 3:**
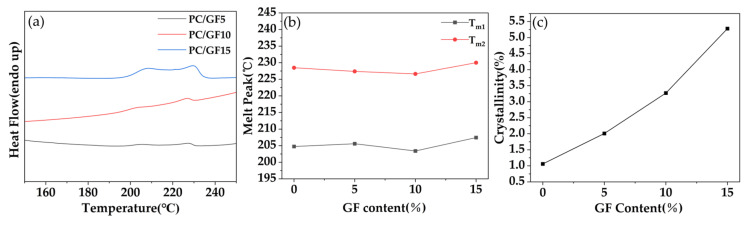
(**a**) DSC graph, (**b**) melting peak temperature graph and (**c**) variation of crystallinity graph.

Figure 2 shows the DSC curve plot, the melting peak temperature plot, and the variation of crystallinity of PC/GF samples with different GF additions under the saturation conditions including the time of 180 min, the pressure of 10 MPa, and the temperatures of 160 °C. As shown in Figure 3a,b, the DSC curves indicated two endothermic peaks. The low-temperature peak (T_m1_) was located at approximately 205 °C, and the high-temperature peak (T_m2_) was detected at approximately 230 °C. The observed peaks were attributed to the melting of PC crystals [36,37] that had formed during the SC-CO_2_ foaming process. These two melting peaks became more obvious with the increase in GF content, but the temperature of the melting peaks almost did not differ, which indicates that the increase in GF content promoted the crystallization behavior. According to Figure 3c, it can be seen that the crystallinity increases with the increase in GF content. The investigation of PC compounded with various fillers under SC-CO_2_ conditions has yielded insightful results. Specifically, in the study conducted by Gedler et al. [38], the inclusion of GnP in PC induced a greater degree of crystallization. This observation was similarly reported by Lai et al. [26] in their investigation of PC compounded with CNTs under SC–CO_2_ conditions, where the addition of CNTs promoted crystallization of the PC matrix foam. These findings suggest that the incorporation of certain fillers in PC under SC-CO_2_ conditions induces increased crystal formation, a phenomenon attributed to the fillers’ ability to induce heterogeneous nucleation. This, in turn, accelerates the PC foaming process, ultimately leading to a greater quantity of crystals.

#### 3.3.2. Effect of Saturation Time on Crystallization Behavior

**Figure 4:**
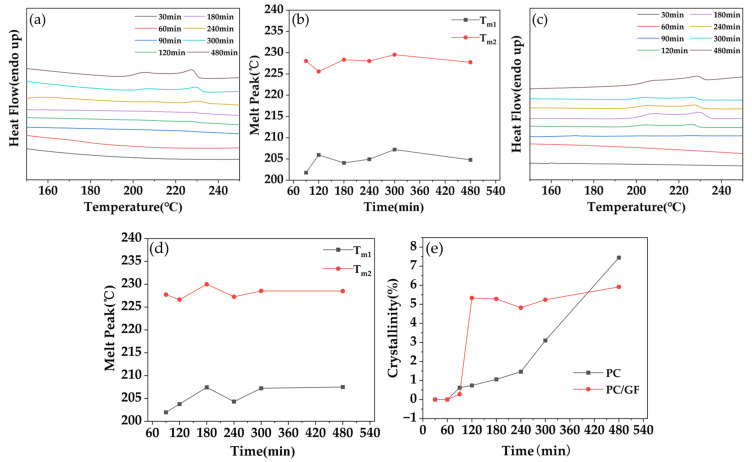
(**a**,**b**) DSC plots and melt peak variation plots of PC, (**c**,**d**) DSC plots and melt peak variation plots of PC/GF15, (**e**) variation plots of crystallinity of PC and PC/GF15.

PC and PC/GF15 composites were treated with different saturation times, and their temperature and pressure were kept at 160 °C and 10 MPa. The changes in crystallization behavior of the samples were observed by DSC test. As shown in Figure 4a, when the saturation time was 90 min, two crystallization peaks appeared on the DSC curve, distributed around 205 °C and 225 °C, and the crystallization peaks at both positions increased with the increase in foaming time, and the enthalpy change of the former crystallization peak was always smaller than that of the latter one. There was no obvious pattern in the temperature of the melt peak with the increase in foaming time. From Figure 4e, it can be seen that the crystallinity was 0.62% at 90 min, and 1.06% at the foaming time from 90 min to 180 min, which indicated the crystallinity did not have obvious change. Increasing time resulted in a significant increase in crystallinity. That is, when the time was 480 min, the crystallinity reached 7.45%. As can be seen from Figure 4c, when the foaming time was 90 min, a slight crystallization phenomenon appeared in the PC/GF15 combined material. It is consistent with the crystallization phenomenon of pure PC, where two crystallization peaks appeared, probably due to the generation of the earliest batch of crystals, which takes some time and is not affected by the addition of GF. When the time was further increased to 120 min, the crystallization phenomenon of the composites occurred obviously, and there was no obvious change in the crystallinity with the increase in time. This indicated that the addition of GF accelerated the formation of crystallization after the earliest batch of crystallization and perfected the crystal structure in a short time, and there was no obvious change in the crystallinity. The reason is that GF induces heterogeneous nucleation to make the foaming process more effective and, at the same time, creates more possibility for the formation of the binding of PC molecular chains and thus generates more crystals in a shorter time. However, at 480 min, the crystallinity of PC is higher than that of PC/GF15 composite. The reason for the analysis is that GF does not produce crystallization in the PC matrix, and the presence of GF hinders the growth space of crystals, making the crystallinity lower. Lai et al. [26] found in their study of composite foams of PC/CNTs that the addition of CNTs resulted in a faster attainment of maximum crystallinity for PC compared with pure PC. However, the extent of the difference in crystallinity between the two was not specified in their results.

#### 3.3.3. Effect of Foaming Temperature on Crystallization Behavior

**Figure 5:**
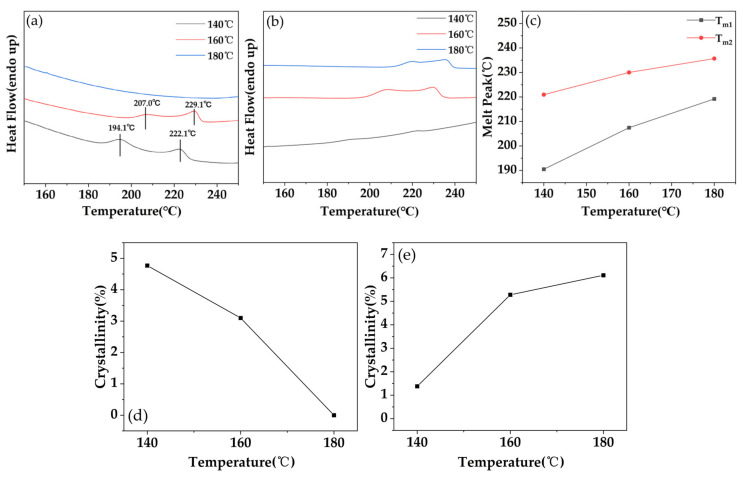
(**a**) DSC plot of PC, (**b**,**c**) DSC plot and melt peak variation of PC/GF15, (**d**) variation of crystallinity of PC, and (**e**) variation of crystallinity of PC/GF15.

Combined with the effect of saturation time on foaming behavior in the previous subsection, a saturation pressure of 10 MPa was chosen, a saturation time of 300 min was chosen for PC, and 180 min was chosen for PC/GF15, and the samples were foamed at different temperatures. Figure 5a shows the DSC plots of pure PC at different temperatures, showing the variation in crystallization peak temperature and crystallinity. Two crystallization peaks appeared at 140 °C and 160 °C, but no crystallization peak appeared at 180 °C. Compared with 160 °C, the temperatures of both crystallization peaks were lower at 140 °C. The proportion of the melting peak where T_m1_ was located was higher at 140 °C, indicating that the proportion of T_m2_-related crystallization was increasing with the increase in temperature. As can be seen in Figure 5d, the degree of crystallinity decreased from 4.77% to 3.1% from 140 °C to 160 °C, and no crystallization was produced at 180 °C, indicating that the degree of crystallinity decreases with increasing temperature. Similar results were obtained by LIAO et al. [39], and the reason for no crystallization at 180 °C may be that when the temperature is too high, the chain segment activity is too large and tends to be disordered and chaotic. It is difficult to orderly arrange the crystal formation. As can be seen in Figure 5b,c, the DSC plot of PC/GF15 has two melt peaks at each temperature condition. As the temperature increases, the two melt peaks get closer to each other and move to higher temperatures. The percentage of T_m2_-related crystallization also gradually increases with increasing temperature, which indicates that more stable crystals are formed as the temperature increases, as shown in Figure 5e. There is a clear difference with the experimental results of PC. The addition of GF increased the viscosity of the composite. Under high viscosity conditions, the increase in temperature decreased the viscosity and enhanced the mobility of the chain segments, which were more likely to form perfect crystals due to the alignment.

### 3.4. Foaming Performance of PC and PC/GF Composites

#### 3.4.1. Effect of GF Content on Foaming Behavior

**Figure 6:**
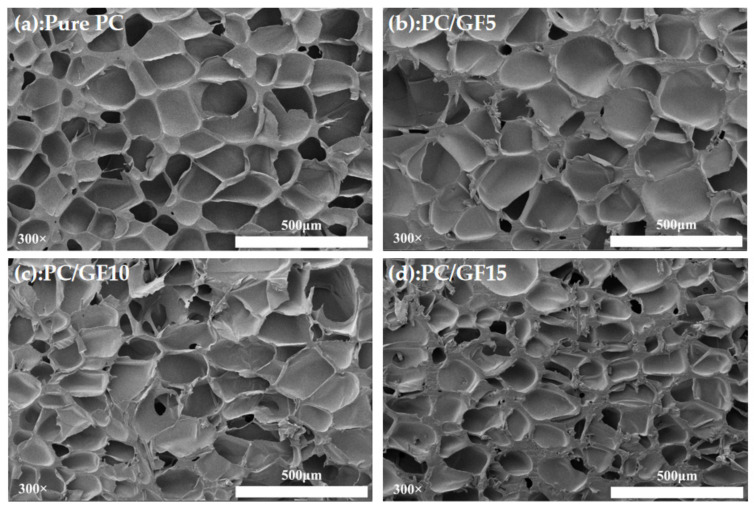
SEM micrographs of foamed PC and PC/GF composites with the GF loading (wt%). (**a**) 0%, (**b**) 5%, (**c**) 10%, (**d**) 15%.

In order to study the effect of GF content on foaming behavior, PC/GF composites with pure PC and GF contents of 5%, 10%, and 15% were foamed under the conditions of saturation pressure of 10 MPa, foaming temperature of 160 °C, and holding time of 30 min. Figure 6 shows the cell morphology. The increase in GF content could decrease the number of cells diameter unit area and increase the number of cells. Under the condition of 15% GF, the bubble wall thickens obviously, which indicates that the viscosity is increased with the addition of GF. These results suggest that GF can significantly affect the foaming behavior of PC by altering its viscosity, as supported by rheological performance tests. In a related study, Wang et al. [40] investigated the effect of GF on the foaming behavior of PLA/GF composites prepared under SC-CO_2_ conditions. It was found that the addition of GF increased the viscosity of PLA, resulting in improved bubble morphology and more regular and uniform bubble characteristics. In contrast, severe bubble collapse and agglomeration were observed during the foaming of pure PLA. These findings suggest that GF can modify the foaming behavior of PLA by increasing its viscosity, which is similar to the behavior affecting PC foaming. Ma et al. [41] utilized SC-CO_2_ to fabricate microporous foam of phenylene sulfide (PPS)/GF composite. The incorporation of GF led to an increase in the viscosity of the PPS/GF matrix. However, it resulted in a gradual increase in the diameter of the composite foam bubbles. The analysis revealed that the bubble diameter in the PPS/GF composite foam, prepared by Ma et al., was considerably smaller (2–7 μm) than the size of GF. As a result, GF was unable to induce PPS heterogeneous nucleation, and the solubility of the PPS matrix was reduced significantly. This further reduced the degree of foaming, leading to a decrease in the number of bubble pores formed. The limited diffusion of CO_2_ gas into a restricted number of bubble nucleation sites resulted in the formation of relatively large bubble structures.

**Figure 7:**
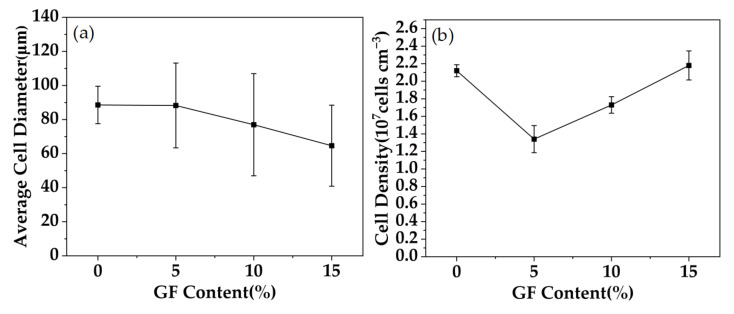
Foam PC and PP/GF composites under different GF loads: (**a**) changes in average cell diameter and (**b**) cell density.

Figure 7 shows the variation of cell diameter and cell density with GF content. The cell diameter of PC composites with pure PC and 5% GF content has little difference, but the cell density decreases obviously. When the GF content continues to increase, the cell diameter becomes smaller and smaller, while the number of cells obviously increases. The reason is that when the content of GF is low, the addition of GF plays a leading role in the decrease in the solubility of CO_2_, reduces the degree of foam supersaturation, and increases the number of formed bubble nuclei. CO_2_ diffuses into the limited bubble nuclei, and the bubble cells will become larger. When the content of GF continues to increase, a large number of GF provide heterogeneous nucleation points, which significantly increases the number of cells, but the solubility of CO_2_ will further decrease, resulting in a decrease in the degree of foaming. The addition of GF will increase the viscosity of PC, restrict cell growth, and finally form a foam structure with a thick running wall and small cells.

#### 3.4.2. Effect of Saturation Pressure Content on Foaming Behavior

**Figure 8:**
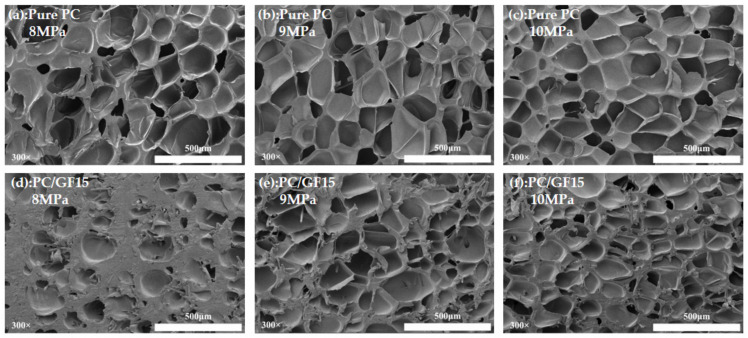
SEM micrographs of foamed PC and PC/15 % GF at different saturation pressure. PC (**a**) 8 MPa, (**b**) 9 MPa, (**c**) 10 MPa. PC/15%GF (**d**) 8 MPa, (**e**) 9 MPa, (**f**) 10 MPa.

Figure 8 is the SEM diagram of the cell morphology of PC and PC/GF15 under the conditions of saturation time of 30 min, foaming temperature of 160 °C, 8 MPa, 9 MPa, and 10 MPa pressure. It can be seen from the figure that PC can complete foaming under conditions of 8–10 MPa. Increasing the pressure makes the cell size get smaller and increases the number of cells. However, the number of cells in PC/GF15 under an 8 MPa condition is very small due to the low CO_2_ solubility of the composite itself, and the low pressure reduces the solubility of CO_2_, which leads to a small number of cells. With the increase in pressure, the number of bubbles per unit area increases gradually. Ma et al. [41] observed a similar trend in their study, using SC-CO_2_ to prepare microporous foams of PPS and PPS reinforced with GF composites. Their findings showed that the number of bubble pores in both pure PPS foams and PPS/GF composite foams increased with increasing pressure.

**Figure 9:**
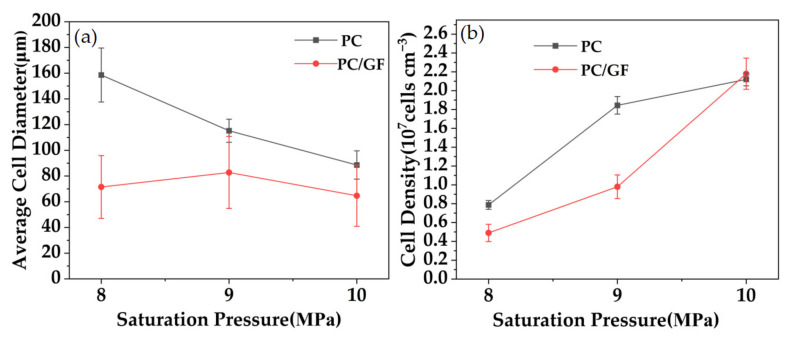
Pure PC and 15% GF loaded PC/GF at different pressures: (**a**) changes in mean cell diameter and (**b**) changes in cell density.

Figure 9 shows the variation of cell diameter and cell density with pressure for PC and PC/GF15 under the conditions of 30 min and foaming temperature 160 °C. With the increase in pressure, the cell diameter of PC decreases gradually, but the cell diameter of PC/GF increases at first and then decreases, and the cell density increases gradually. The reason is that the solubility of CO_2_ in the PC matrix increases, which reduces the free energy for the matrix to form bubble nuclei, forms more bubble nuclei, and increases the cell density. However, with the increase in pressure, too many bubble nuclei make the space for cell growth smaller, and thus the cell diameter becomes smaller. Compared with PC/GF at 8 MPa, too low a CO_2_ solubility and too high a viscosity hindered the foaming behavior. When the pressure was further increased, the foaming degree increased and the cell diameter increased. When the pressure is 10 MPa, more foam is formed, which makes the foaming space smaller and the cell diameter smaller. The cell diameter of PC/GF is always smaller than that of PC, which is due to the increase in viscosity of the PC matrix caused by GF, which hinders cell growth and decreases the cell diameter. The cell density of PC/GF is affected by viscosity at low pressure; the cell growth is blocked, and the cell density is lower. When the pressure increases and the pressure drop increases, the critical free energy for cell nucleation decreases, and heterogeneous nucleation results in more cell structure.

#### 3.4.3. Effect of Foaming Temperature on Foaming Behavior

**Figure 10:**
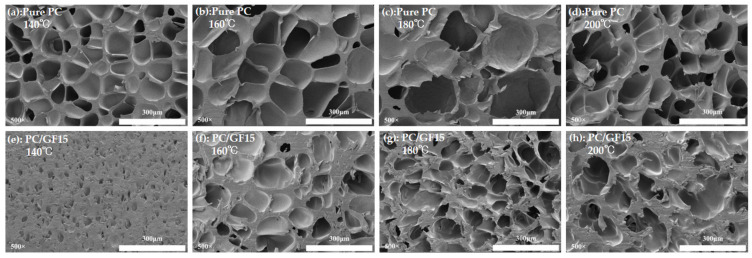
SEM micrographs of foamed PC and PC/15 % GF at different foaming temperatures. PC (**a**) 140 °C, (**b**) 160 °C, (**c**) 180 °C, (**d**) 200 °C. PC/15%GF (**e**) 140 °C, (**f**) 160 °C, (**g**) 180 °C, (**h**) 200 °C.

The effect of foaming temperature on the cell morphology of PC and PC/GF15 composite microcellular materials is shown in Figure 10. All the samples were carried out under the conditions of pressure 10 MPa, heat preservation, and pressure preservation for 30 min, and the foaming temperature was 140, 160, 180, 200 °C. According to the SEM diagram, the cell size of PC increases gradually with increasing the temperature from 140 °C to 180 °C, and the bubble wall becomes thinner. When the temperature rises to 200 °C, the bubble wall is obviously broken and the cells are connected. This is because with the increase in temperature, the viscosity of the PC matrix decreases, the resistance to cell growth decreases, and the cell becomes larger. At 200 °C, the viscosity of the PC matrix is too low, which leads to the phenomenon of bubble wall rupture and connectivity. However, because the temperature of PC/GF is too low and the viscosity of the composite itself is too high at 140 °C, the viscosity of the composite is too high, and the cell growth is blocked, which is obviously different from the simple PC. When the temperature is further increased by 180 °C, the viscosity of the material decreases, the cell growth resistance decreases, and the bubble wall becomes thinner. When the temperature is 200 °C, the adjacent bubble cells are broken and connected, but the bubble walls become thicker. The reason is that too high a temperature leads to a decrease in the solubility of CO_2_ in the composites, resulting in the formation of fewer bubble nuclei, while between adjacent bubble nuclei, the bubble walls break and merge because of low viscosity. Similar results were obtained by Ma et al. [16] for the preparation of PC microporous foam using SC-CO_2_.

**Figure 11:**
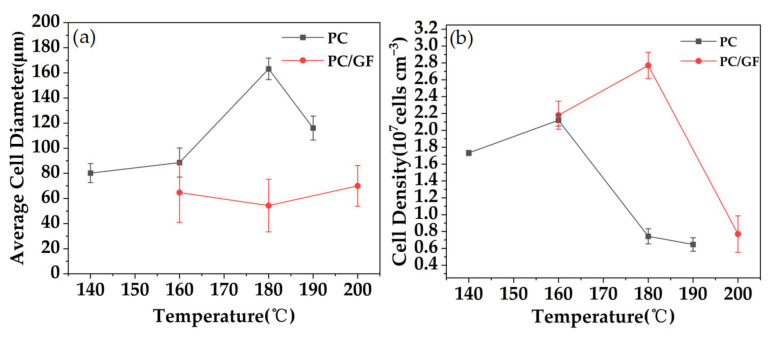
Pure PC and 15% GF loaded PC/GF at different foaming temperatures: (**a**) changes in mean cell diameter and (**b**) changes in cell density.

Figure 11 shows the variation of cell diameter and cell density in PC and PC/GF with different pressures. For PC, with the increase in temperature, the viscosity of the matrix decreases gradually, the resistance of cell growth decreases, the cell produced by foaming becomes larger, and the bubble wall has been slightly damaged at 180 °C. When the temperature reaches 200 °C, the viscosity of the matrix is too low, and the cell wall is broken to make the cells connect. The ruptured channel leads to the loss of CO_2_, the difficulty of cell growth, and the decrease in cell diameter. The cell density increases at this stage, and the reason is that compared with the decrease in CO_2_ solubility caused by the increase in temperature, the thermodynamic instability caused by the increase in temperature is dominant, which leads to the higher degree of foaming and the increase in cell density. When the temperature increased to 160–200 °C, the relative density and cell density decreased because the high temperature led to a decrease in the solubility of CO_2_ in the PC matrix, resulting in a lower foaming degree and lower foam density. The decrease in solubility results in the decrease in bubble nuclei and cell density during foaming. For PC/GF, the cell diameter decreased at first and then increased, and the cell density decreased at first and then increased. The reason is that at 180 °C, the super-saturation of the foaming system is larger and the thermodynamic instability is more obvious, which leads to a decrease in the viscosity of the matrix material, a decrease in the resistance to the formation of bubble nuclei, and an increase in cell density. The higher cell density limits the growth space between the cells and reduces the cell diameter. When the temperature increased to 200 °C, the viscosity of the matrix continued to decrease, and the outward diffusion of CO_2_ became the key to nucleation, while too high a temperature decreased the solubility of CO_2_ and the cell density. In addition, due to the decrease in viscosity caused by high temperature, the cell growth resistance decreased and the cell size increased. The cell diameter of PC/GF is always smaller than that of PC under the same conditions, and the cell density is always higher than that of PC, which is mainly attributed to the effect of GF on the viscosity of PC and heterogeneous nucleation induced by GF.

#### 3.4.4. Effect of Saturation Time on Foaming Behavior

**Figure 12:**
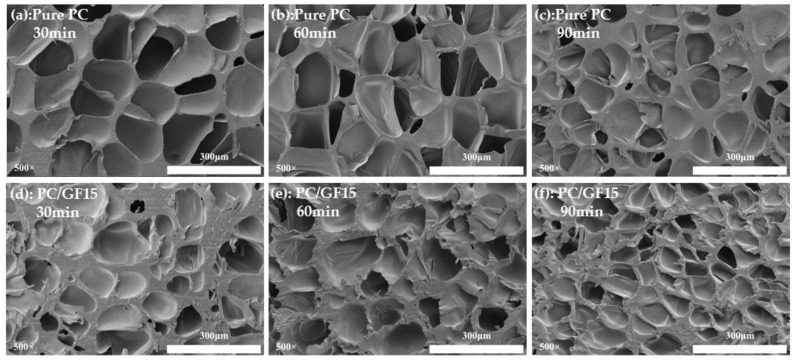
SEM micrographs of foamed PC and PC/15 % GF at different saturation times. PC (**a**) 30 min, (**b**) 60 min, (**c**) 90 min. PC/15%GF (**d**) 30 min, (**e**) 60 min, (**f**) 90 min.

Figure 12 shows the SEM diagram of cell morphology of PC and PC/GF15 foaming materials with different saturation time under the foaming temperature of 160 °C and the pressure of 10 MPa. For PC foaming materials, when the holding time increases from 30 min to 60 min, the cell morphology changes slightly, but when it continues to increase from 60 min to 90 min, the cell becomes smaller and the cell wall becomes thicker. The reason for the analysis may be that the time from 30 min to 60 min increases the solubility of CO_2_ in the matrix and makes the foaming degree better. When the time increases to 90 min, more CO_2_ dissolved in the matrix causes the polymer chain in the PC matrix to relax and crystallize. In a certain time, the crystallization induced by CO_2_ will form the nucleation point of the cell and increase the cell density, but the crystallization will discharge the CO_2_ dissolved in the PC matrix, which will hinder the foaming behavior, make the cell smaller, and thicken the cell wall. For PC/GF15 foaming materials, the change trend of cell morphology remains constant, but the number of cells in 90 min is significantly higher than that in PC. Mohammadreza et al. [42] obtained similar results for microporous foaming of thermoplastic polyurethane (TPU) under SC-CO_2_ conditions.

**Figure 13:**
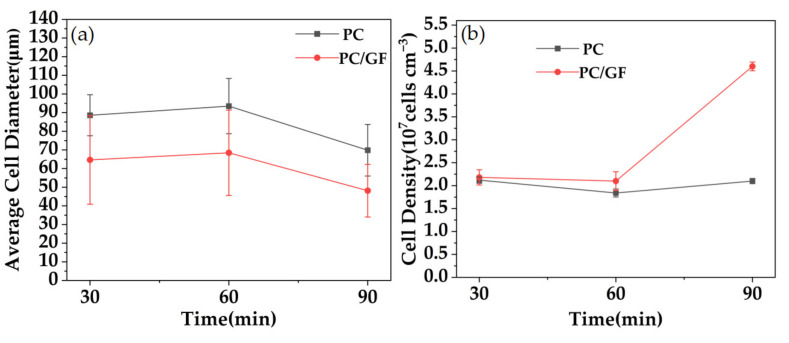
Pure PC and 15% GF loaded PC/GF at different saturation times: (**a**) changes in mean cell diameter and (**b**) changes in cell density.

Figure 13 shows the relative densities, diameters, and densities of microcellular foams of PC and PC/GF15 foaming materials at different temperatures. The cell diameter and cell density are consistent, and there is no obvious change from 30 min to 60 min. When increasing to 90 min, the cell diameter decreases because of the limited foaming degree, and the cell density of PC/GF foaming material increases obviously due to crystallization and heterogeneous nucleation of GF, reaching 4.6 cells/cm^3^.

### 3.5. Thermal Performance Analysis of PC Foam and PC/GF Composite Foam

**Figure 14:**
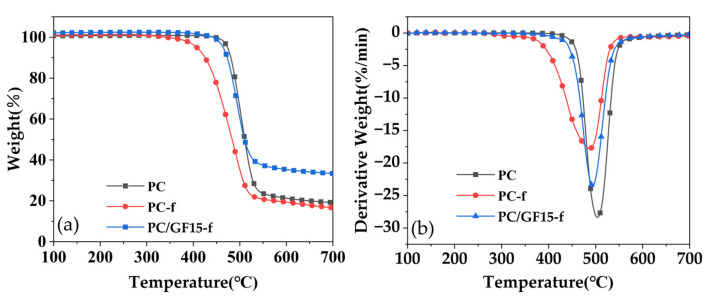
(**a**) TGA mass weight loss curves and (**b**) DTG curves for PC, PC-f and PC/GF15-f composites.

PC foamed materials (PC-f) and PC/GF15 foamed materials (PC/GF15-f) with low relative densities were selected for thermal performance analysis. Figure 14 shows the thermogravimetric curve (TGA) and derivative thermo-gravimetric (DTG) of PC, PC-f and PC/GF15-f in a nitrogen atmosphere. The start of decomposition temperature and the maximum decomposition temperature of PC-f were both earlier, at 384 °C and 487 °C, respectively, which may be because foaming changes the molecular chain activity of PC and leads to the advancement of the initial decomposition temperature, and the weight loss rate decreases with the increase in cell structure. The initial decomposition temperature of PC/GF15-f is close to that of pure PC and much better than that of PC-f, which is due to the flame-retardant effect of glass fiber, which prevents the material from decomposing in advance and improves the thermal stability of the material. The decomposition behavior of all kinds of PC material is one-step decomposition, but the carbon residue rate of PC/GF15-f at 700 °C has been greatly increased, which is due to the residue of undecomposed glass fiber. From the DTG curve, it can be seen that the curve of weight loss rate of PC/GF15 foaming material is close to that of pure PC, and the maximum decomposition rate of PC foaming material is lower than that of pure PC foaming material. The reason may be related to the dispersion of glass fiber and the change in cell structure.

**Figure 15:**
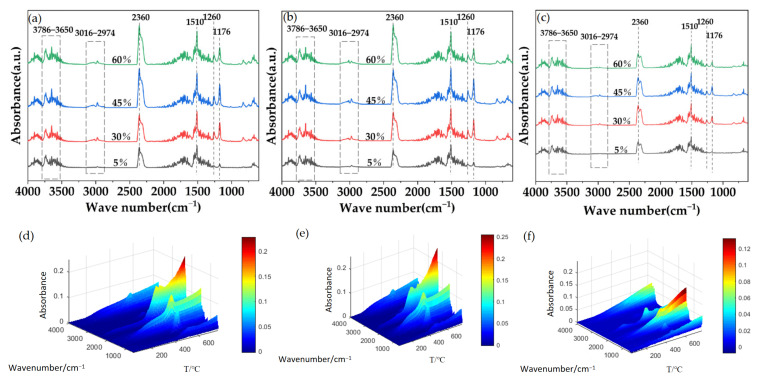
Infrared spectra of (**a**) PC, (**b**) PC-f and (**c**) PC/GF15-f gas phase cleavage products, 3D IR spectra of (**d**) PC, (**e**) PC-f and (**f**) PC/GF15-f gas phase cleavage products.

To further investigate the reasons for the altered decomposition behavior of PC, PC-f, and PC/GF15-f, the gas-phase cracking products of the composite foams were analyzed using TGA-FTIR in conjunction. Figure 15 shows the infrared spectra of the gas-phase cracking products of PC, PC-f, and PC/GF15-f at 5%, 30%, 45%, and 60% mass loss. It can be found that the main characteristic peaks are basically the same: 3786–3650 cm^−1^ for H_2_O; 3016–2974 cm^−1^ for the stretching vibration of alkane C-H; 1510 cm^−1^ for the backbone vibration of phenyl compounds; 1260 cm^−1^ for the stretching vibration of aromatic ether C-O; and 1176 cm^−1^ for the stretching vibration of C-OH [43]. On the other hand, the peak intensity of PC/GF15-f at all stages is always lower than that of pure PC and PC foams, which is attributed to the barrier effect of the glass fibers preventing the thermal degradation process. Therefore, it can be found that the addition of glass fiber does not change the cleavage process of the PC molecular chain and that the glass fiber plays a certain role as a flame retardant in the composite foaming material, which makes the maximum decomposition rate lower. However, the volume of the material increases after foaming, and the dispersion of glass fiber occurs, which makes the flame-retardant effect worse. After the addition of GF, the thickness of the walls surrounding the bubble holes increases, and the regularity and compactness of the bubble holes decrease. This is the primary reason why the maximum decomposition rate of PC/GF composite foams is higher than that of pure PC. Jang et al. [43] analyzed the cleavage products of PC using TGA-FTIR and obtained similar results.

## 4. Conclusions

The PC/GF composites were prepared by the extruding process. The PC/GF composite foams were prepared by using the batch foaming process with SC-CO_2_ blowing agent. The following conclusions were drawn:

- The molecular structures of PC/GFs with physically doping were not changed analyzing by the infrared spectra. The different “soaking” periods within the autoclave resulted in different microcellular structures of PG foams. The addition of GFs can accelerate the crystallization stability of PC induced by SC-CO_2_ and form a large number of crystals in a short time.
- The addition of GFs increases the viscosity of PC and the thickness of the bubble wall. The GF additive, as heterogeneous nucleation, can greatly increase the cell density to 4.6 cells/cm^3^. The Thermo-gravimetric analysis (TGA) results show that the PC/GF foams have a significant increase in the residual carbon rate at 700 °C. The weight loss rate was less than that of PC matrix.
- The TG-FTIR connection testing results explain that the addition of GFs did not change the pyrolysis products of PC/GF composites, but decreased the cracking strength and improved the thermal stability. This study showed that adding GF to PC foams could significantly improve the thermal stability and crystallization properties of PC/GF composites.
- PCFs are already being used in the construction industry, automotive interiors, and medical devices. The research results provide useful guidelines on industrial production and applications of PCFs. The fracture mechanism of the tough-brittle transition of PCFs needs to be further investigated.

## Data Availability

The data presented in this study are available on request from the corresponding author.
